# The International Medical Congress

**Published:** 1885-08

**Authors:** 


### Editorial.

### THE INTERNATIONAL MEDICAL CONGRESS.

A family quarrel has grown out of the arrangements on the part of the American Medical Association in regard to the meeting of the International Medical Congress in 1887, and matters are getting into a muddle in this otherwise well regulated household. It appears that some time ago, certain members of the family were authorized to invite guests to a feast, who did not observe exactly the style that was considered proper by the head of the establishment, and hence, others were designated to control their future proceedings. This has worked out so as to prove distasteful to those who originally undertook to do the courtesies of the house, and instead of making progress with the service assigned their joint representatives, dissension is likely to impair the provisions for the banquet and mar the enjoyment of the occasion, if not eventually cause disgust to the invited guests so as to prevent their attendance.

We are not exactly prepared to point out the origin of this trouble, yet it must have been observed that the work undertaken by the original committee progressed well until the meeting of the American Medical Association at New Orleans, and that the new order of things instituted then and there has brought about a change for the worse. It is a very safe motto in all business matters to let well enough alone, and although everything had not been done by the representatives of the medical profession in this country precisely as some thought proper, there was no serious departure from that course which was calculated to give effect to the intention of their appointment. That spirit of criticism which overlooks the main end, and cavils at the minor shortcomings in the execution of an important trust, has brought very unsatisfactory results in this matter; and, unless wise counsels shall prevail over the petty jealousies that have been at work recently, a disastrous failure must attend the organization of a medical congress in this country.

That the recent action of the American Medical Association is sustained by parliamentary usage, does not in any way commend the expediency of its course in view of all the facts. An executive committee of seven most assuredly afforded a better prospect of efficiency in making "all necessary and suitable arrangements for the meeting of such Congress" than one of forty-five members, such as provided for, with the addition of thirty-eight to the original committee under the resolutons of 1884.

But the practical question that presses itself upon the attention of all who have the honor of the medical profession at heart, is to provide a mode of escape from the difficulties surrounding this issue of personal relations rather than professional associations.

As the invitation emanated from the American Medical Association, it is evident that its representatives in conducting the business must follow the programme laid out by that body, and as a number of the committee do not see their way clear in this respect, so that their resignations have been tendered, the only alternative left is that the provisions made for supplying their places shall be adopted, thus completing the number requsite for the transaction of all that pertains to the organization of the congress upon a proper basis.

